# Transitional Cell Carcinoma of the Upper Ureter Metastatic to the Thoracic Spine Presenting as a Spinal Cord Compression

**DOI:** 10.1100/tsw.2008.43

**Published:** 2008-02-25

**Authors:** J. O. Larkin, I. M. Cullen, M. O. Kelleher, J. Fitzgibbon, K. Keohane, M. G. J. O'Sullivan, E. Rogers

**Affiliations:** ^1^ Department of Urology, Mercy University Hospital Cork, Ireland; ^2^ Department of Neurosurgery, Cork University Hospital, Ireland; ^3^ Department of Pathology, Mercy University Hospital Cork, Ireland; ^4^ Department of Pathology, Cork University Hospital, Ireland

**Keywords:** Transitional cell carcinoma, metastatic, spinal cord compression

## Abstract

We performed a left nephroureterectomy for a gentleman with transitional cell carcinoma of the upper ureter. Histological analysis revealed it to be a T1 lesion, but to be highly mitotically active. The gentleman defaulted on adjuvant therapy and defaulted on follow-up. He represented with symptoms of acute spinal cord compression and magnetic resonance imaging demonstrated a lesion at T6/7. Neurosurgical resection of the lesion showed it to be a metastatic deposit from the ureteric primary. Despite surgical debulking and subsequent radiotherapy to the lesion, the patient died secondary to metastatic complications. This case report is of interest to the surgeon as it demonstrates both the high metastatic potential of upper tract carcinomas and educates the surgeon on the presentation of acute spinal cord compression.

## INTRODUCTION

Transitional cell carcinoma (TCC) is significantly more common in the lower than in the upper urinary tract. We report an unusual presentation of metastatic TCC from the upper tract urothelium to the thoracic spine following surgical resection of the primary lesion. It resulted in a typical spinal cord compression presentation.

## CASE REPORT

A 60-year-old man presented to our department with a 6-month history of intermittent frank painless haematuria. History, physical examination, and biochemical investigations were unremarkable. Cystoscopy showed a normal bladder. Subsequent retrograde pyelography identified a 2.5-cm lesion in the upper left ureter with mucosal destruction.

A left nephroureterectomy was performed. Histological analysis revealed a grade 3 (poorly differentiated) papillary TCC. The tumour was highly mitotically active without evidence of necrosis and surgical margins were clear, representing stage 1 (mucosal lamina propria involved with T1/N0/M0) cancer of the ureter. Computed tomography of the abdomen and pelvis performed to stage the disease showed no extraureteric spread. The patient made an uneventful postoperative recovery, but refused adjuvant chemotherapy and defaulted on out-patient follow-up.

The patient returned 12 months later with a 6-day history of back pain, progressive lower limb weakness, constipation, and finally urinary retention. Examination elicited hypertonicity, hyper-reflexia, and spastic paralysis of the lower limbs with upper limb sparing, and a sensory level at T5. Total collapse of the left T5 pedicle was demonstrated on plain radiography and the patient was transferred to the department of neurosurgery. MRI showed a large mass lesion to the left of the T6/7 vertebra involving the pedicle and lamina, and grossly compromising the cord (Figures 1 and 2).

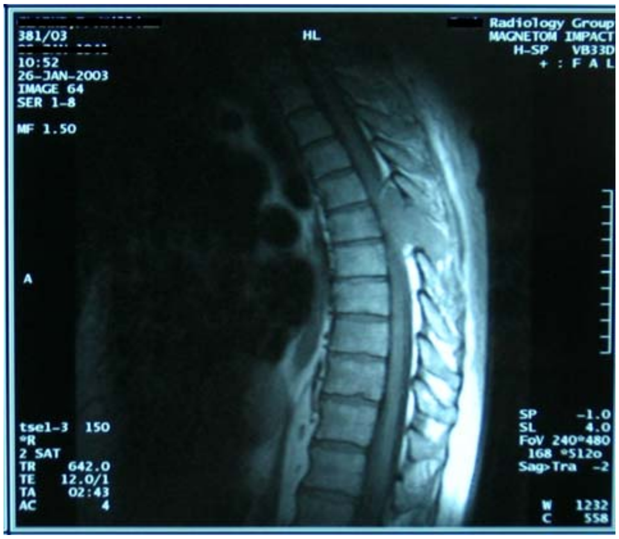

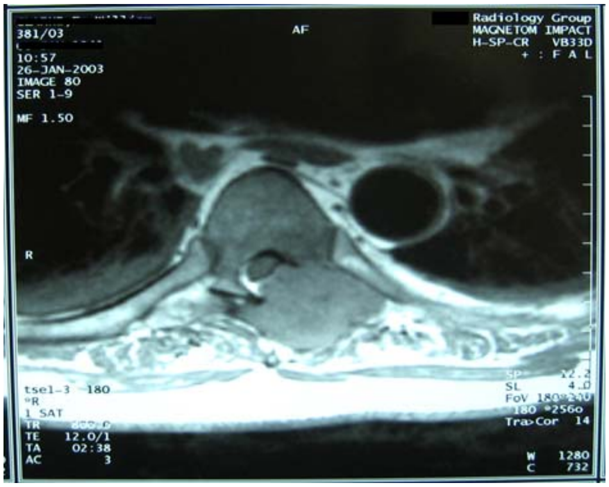

**FIGURES 1 and 2.** T1 weighted saggital (1, left) and axial (2, right) images demonstrating a mass lesion of intermediate signal intensity at T6/7 level. The lamina and pedicle are involved at the T6 level and the spinal canal is grossly compromised, displacing the cord laterally.

The patient underwent laminectomy and debulking of the tumour. Postoperatively, he received radiotherapy, chemotherapy, and intensive physiotherapy, eventually making a partial functional recovery. Histology of the debulked tumour showed metastatic TCC in bone with an osteoblastic response, necrosis and anaplasia (Figures 3 and 4) and a focal papillary pattern, confirming the lesion to be a metastasis from the ureteric primary.

Thereafter, he was sent to the National Rehabilitation Hospital for physiotherapy and rehabilitation. However, there he developed progression of his paraplegia and MRI revealed recurrence of the tumour mass with spinal cord compression at T6 and T7 level. This was further treated with radiotherapy, but there was no return of power and the patient had a resultant paraplegia with loss of sphincteric control. He was subsequently admitted to a palliative care institution where he died secondary to respiratory compromise.

## DISCUSSION

TCC of the upper urinary tract is an uncommon disease, accounting for only 4.5–9% of all renal tumours and 5–6% of all urothelial tumours. Nephroureterectomy with bladder cuff removal has been considered the standard treatment for upper urinary tract tumours due to the high recurrence rate in the remaining distal ureter (16–58%), multiplicity of urothelial tumours in the same voiding system (27–36%), and low incidence of bilateral tumours (2–8%)[1,2].

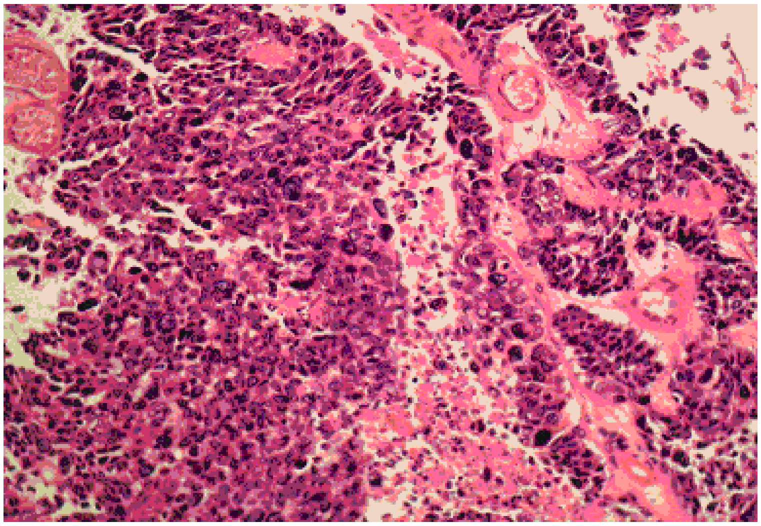

**FIGURE 3.** 200X H&E stained TCC demonstrating widespread necrosis and anaplasia

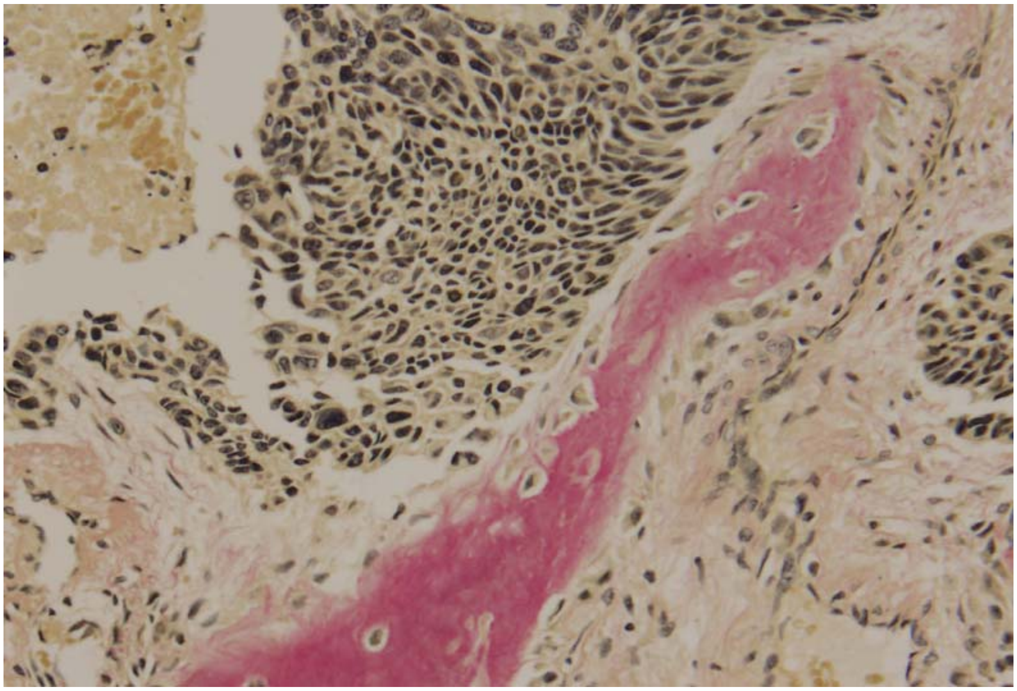

**FIGURE 4.** Central fragment of newly formed bone (pink) immediately surrounded by a layer of osteoblasts and with sheets of TCC replacing the marrow.

Tumour recurrence and progression rates have been shown to be higher in patients with high-grade tumours or tumours with lamina propria invasion[2]. Initial reports indicate that patients with urothelial TCC at high risk of relapse after radical surgery can have a reasonable chance of long-term survival with systemic adjuvant chemotherapy, although the full benefits of such treatment remain to be elucidated in a large randomized controlled trial[3].

Despite some of the controversies in local control of the upper tract TCC after surgery by adjuvant radiation therapy, almost all of the clinical studies stress the ineffective nature of this approach in prevention of sytemic disease and subsequent death[4].

In a series of 26 patients with stage T3 urothelial carcinoma of the renal pelvis or ureter, Cozad et al.[5] treated 17 patients with surgery only and nine received adjuvant radiation therapy (median dose: 50 Gy) in an effort to enhance the local control of the disease. There was no difference in local failure either with or without adjuvant radiation in low-grade tumours. However, high-grade lesions had a local failure rate of 15% with and 71% without adjuvant radiation therapy. The authors concluded that high-stage urothelial carcinoma of the upper urinary tract had a substantial local failure risk after surgery alone. Adjuvant radiation therapy markedly reduced this risk, but had no impact on distant disease, which occurred in approximately 50% of the cases[5].

A large European multicentre retrospective series looking at 138 patients with TCC of the renal pelvis and/or ureter showed a 5-year loco regional recurrence rate of 62% in patients treated with nephroureterectomy without adjuvant therapy[6]. However, the authors could not show any benefit of postoperative radiation therapy. Overall survival rates at 5 and 10 years were 21 and 13% in patients with radiation, and 33 and 24% without radiation, respectively.

The absence of any survival benefit by adjunctive radiotherapy certainly indicates the immediate need for an effective systemic therapy to prevent distant failure and related death in this patient population.

Combined radiation and cisplatin-based chemotherapy have proved effective in muscle-invasive bladder carcinoma, and a similar benefit may be possible with upper tract urothelial carcinoma. Czito et al. retrospectively reviewed the records of 31 patients who underwent nephroureterectomy followed by adjuvant radiotherapy with or without concurrent chemotherapy[7]. They found that the addition of concurrent cisplatin to adjuvant radiotherapy improved the ultimate outcome in patients with resected, locally advanced upper tract urothelial malignancies and concluded that this regime should be considered in patients with T3/4 and/or node-positive upper tract TCC.

Similarly Kwak et al. retrospectively evaluated the efficacy of adjuvant systemic chemotherapy in conjunction with surgery in 43 patients with invasive TCC of the upper urinary tract. The results demonstrated that adjuvant systemic MVAC (methotrexate, vinblastine, adriamycin, and cisplatin) could provide therapeutic benefit[8].

Despite the infrequent nature of the disease and obvious disadvantage of a small number of cases in each particular clinical series, evidence emerging from the literature indicates that systemic neoadjuvant[9] and adjuvant chemotherapy may prevent progression to metastatic disease and prolong survival in upper tract urothelial carcinoma.

Computed tomography (CT) imaging is useful in the diagnosis of renal urothelial tumours. It can distinguish between radiolucent renal stones and upper-tract urothelial tumours, since stones appear opaque on CT scans. CT can also be used for determining local extent and distant metastases, but it is of limited value in predicting the pathologic stage of upper-tract urothelial tumours and has a tendency to understage the lesion (accurate in 43–77% of cases)[10,11].

Tumour grade, pathological stage, and vascular invasion are significantly important prognostic parameters in patients with upper urinary tract cancers[12]. Although this tumour was staged pT1, it was a grade 3 lesion and the pathologists reported on the high mitotic activity of the neoplastic cells. Traditionally, adjuvant chemotherapy would be recommended for those with muscle-invasive disease, however in this case, considering the high grade of the neoplastic lesion, we felt that adjuvant chemotherapy may provide a disease-specific survival benefit. For high-grade and high-stage tumours, effective adjuvant treatments along with aggressive surgery may be considered[13].

As with TCCs of the bladder, the most common sites of haematogenous metastasis from the ureter are the lung, liver, and bone[14]. We report the first case, to our knowledge, of isolated thoracic spinal metastasis from a proximal ureteric TCC presenting as spinal cord compression.
